# The effect of resilience training with mHealth application based on micro-learning method on the stress and anxiety of nurses working in intensive care units: a randomized controlled trial

**DOI:** 10.1186/s12909-024-05427-w

**Published:** 2024-04-24

**Authors:** Maryam Abbasalizadeh, Zahra Farsi, Seyedeh Azam Sajadi, Afsaneh Atashi, Andrew Fournier

**Affiliations:** 1 Critical Care Nursing Department, School of Nursing, Aja University of Medical Sciences, Tehran, Iran; 2 Medical-Surgical Nursing, Research and Ph.D. Nursing Departments, School of Nursing, Aja University of Medical Sciences, Kaj St., Shariati St., Tehran, Iran; 3 Nursing Management Department, School of Nursing, Aja University of Medical Sciences, Tehran, Iran; 4 Department of Psychology, Central Branch, Islamic Azad University, Tehran, Iran; 5 Faculty of Psychology, Bangalore University, Bangalore, India; 6 Grand Canyon University, Arizona, USA

**Keywords:** Nurses, Micro-learning, mHealth, Intensive care, Resilience, Stress, Anxiety

## Abstract

**Introduction:**

Nurses in intensive care units (ICUs) face high stress and anxiety, impacting their well-being and productivity. Addressing this, this study evaluated the impact of resilience training via a mHealth application based on micro-learning on ICU nurses’ stress and anxiety levels.

**Materials and methods:**

This study, a single-blind randomized controlled trial conducted in 2022-23, involved sixty ICU nurses from two Tehran hospitals. Nurses were chosen through purposive sampling and divided into intervention and control groups by simple randomization. The intervention group was taught resilience via an educational mHealth application based on micro-learning, with data collected using the anxiety and stress subscales of DASS-21.

**Results:**

Before the intervention, there were no significant differences in stress and anxiety scores between the intervention and control groups (*P* > 0.05). Upon utilizing the mHealth application, the intervention group exhibited significant reductions in stress, from 10.77 ± 3.33 to 9.00 ± 1.66 (*P* = 0.001), and in anxiety, from 9.43 ± 3.35 to 7.93 ± 0.98 (*P* < 0.001). In contrast, the control group experienced a slight increase in stress levels, from 10.10 ± 2.19 to 10.73 ± 2.15 (*P* = 0.002), and in anxiety levels, from 9.10 ± 1.63 to 10.23 ± 1.65 (*P* < 0.0001).

**Conclusions:**

The micro-learning-based mHealth application for resilience training significantly reduced ICU nurses’ stress and anxiety, recommending its adoption as an innovative educational method.

**Trial registration:**

The study has been registered in the Iranian Registry of Clinical Trials (No. IRCT20221225056916N1, Date: 04/29/2023).

## Introduction

Intensive care units (ICUs) nurses frequently deal with high levels of stress and anxiety due to a variety of factors, including demanding workloads, difficult circumstances, high inpatient death rates, and physical aspects of the department, like excessive noise and lighting that are beyond standards [1]. Whereas anxiety is frequently the result of numerous uncomfortable sensations and hypothetical situations, stress usually results from outside sources [2]. One of the known negative effects of stress is anxiety. Anxiety can arise from stressful situations that nurses are unable to control. Although addressing the root causes of stress can lessen it, anxiety is more enduring and becomes a normal part of a nurse’s workday [2].

The stress and anxiety inherent in ICUs, exacerbated by the recent years’ coronavirus disease )COVID) pandemic, have diminished the quality of life, mental well-being, and job satisfaction of nurses [3], resulting in stress disorders, anxiety, depression, and job burnout [2]. There is increasing worry that nurses and other healthcare professionals may leave the field due to occupational stressors [4]. Thus, especially in the post-COVID era, it is imperative to put into practice efficient interventions to lessen occupational stress and anxiety and improve the mental health of critical care nurses [3]. To manage stressful situations, hospital nurses must acquire coping mechanisms and constructive adaptation techniques [5]. Resilience as a coping strategy can help individuals to face stressful conditions and save them from pathogenic disorders [6]. It has been determined that resilience shields nurses’ physical and mental health from stress and anxiety [7]. Resilient nurses acquire the skills to overcome personal and professional obstacles and cope more effectively with stress in demanding circumstances [7]. Evidence show educational interventions can strengthen nurses’ resilience [2, 7, 8]. Some researchers suggest that resilience skills training based on Nan Henderson’s Resiliency Training Program can reduce the job stress and anxiety of emergency and intensive care nurses. This program consisted of self‑confidence reinforcement, management of emotions and feelings, coping with stress, anger management, effective communication, problem‑solving, decision‑making, purposeful planning to achieve goals, and foresight [6, 9]. Other study utilized mindfulness, gratitude, self-care, and social support to enhance the resilience of nurses and mitigate their stress and anxiety [10]. Psychological techniques for increasing resilience and reducing stress and anxiety among nurses vary in content, methods, and implementation duration. Researches typically encompasses a blend of cognitive strategies, mindfulness training, and psychological exercises, primarily aiming to augment an individual’s capacity to effectively manage adverse situations and enhance emotional insight. As well as, various modalities, including online [11] and face-to-face training [12], digital applications [13], a combination of face-to-face and online training [14], a combination of online training and application use [15], and toolkit-based training [16] were used in the previous studies. The duration of interventions in the conducted studies ranged from three [12] to 30 h [14]. Challenges such as nurses’ inability to participate in face-to-face training due to extensive work hours, delays in feedback, difficulties in effectively monitoring learners, frequent network disruptions, and the absence of practical clinical education content on online platforms have been identified. Conversely, the potential for evaluative training via digital mobile applications and the rising popularity of digital smartphones have prompted health organizations to adopt mHealth applications [17].

Several strategies, such as micro-learning, are used to improve the effectiveness of mHealth applications [18, 19]. Educational content is delivered through podcasts, video clips, and graphic transcripts in the micro-learning approach [20]. The following are some advantages of microlearning for nurses’ learning productivity: (a) Conceptual learning: By relating academic material to real-world situations, learners can better understand how the program will be used in practice [21]. (b) Integration into the daily workflow: Micro-learning is a great tool for busy healthcare professionals because it lets participants complete actionable questions on their smartphones while going about their daily duties [21]. (c) Spacing and testing: To combat the forgetting curve, micro-learning uses spaced training methods that reinforce information at varying intervals. The testing effect encourages long-term behavior change by actively engaging students and giving them quick feedback [21]. (d) Data-driven approach: To identify skill gaps and inform future healthcare learning and development programs, microlearning gathers data during the learning process [21]. (e) Patient impact: The experience, safety, and results of patients are directly impacted by professional competence. In just a few minutes each day, the micro-learning reinforces important concepts and best practices, improving the patient experience as a whole [21].

In response to the occupational stress and anxiety experienced by ICU nurses, and adjusting to the recent stress and anxiety imposed on nurses during the COVID pandemic, sometimes resulting in persistent symptoms, the implementation of a wide range of educational interventions is necessary. These interventions aim to promote resilience and reduce stress and anxiety, enabling nurses to continue their professional work. Additionally, preparing for the mental and physical challenges of another crisis is crucial. It has been demonstrated that resilience training improves nurses’ mental health and productivity. However, the review showed that most research has concentrated on conventional approaches to resilience education, with scant data on the effectiveness of active digital strategies like micro-learning. Given the significance of building resilience to reduce stress and anxiety among ICU nurses, this study aimed to investigate the impact of a mHealth application based on the micro-learning method on the level of stress and anxiety experienced by nurses in ICUs. Since the implemented micro-learning approach is an innovative digital method utilizing mHealth applications, it should be contrasted with contemporary distance training methodologies. As new remote learning approaches emerged in response to the COVID pandemic, this study’s findings are compared to those of studies conducted during the pandemic era. This comparison aims to provide a clearer understanding of the quantitative and qualitative effects of the micro-learning approach.

## Materials and methods

### Design and setting

This study was a single-blind randomized controlled trial, where the researcher assistant who conducted the coin toss and the statistical analyst were both unaware of the order and type of intervention administered to the two groups. It was registered in the Iranian Clinical Trials System (No. IRCT20221225056916N1, Date: 29/04/2023) and conducted during the 2022-23 period at two selected hospitals in Tehran, Iran. These hospitals comprised a total of four ICUs, within which 250 nurses were employed.

### Participants and sampling

The sample size was also estimated to be about 24 nurses in each group, according to the sample size formula, the mean and standard deviation (SD) reported in the previous study [9], with a 95% confidence interval, and a 80% test power.

$$\eqalign{{\text{n}} & = \frac{{{{\left( {{{\text{Z}}_{1 - \frac{\alpha }{2}}} + {{\text{Z}}_{1 - \beta }}} \right)}^2}\left( {\delta {1^2} + \delta {2^2}} \right)}}{{{{{\text{(}}{\mu _1} - {\mu _2}{\text{)}}}^2}}} \cr & = \frac{{{{{\text{(}}1.96 + 0.84{\text{)}}}^2}{\text{((}}6.7{{\text{)}}^2} + {{{\text{(}}9.8{\text{)}}}^2}{\text{)}}}}{{{{{\text{(}}36.4 - 43.2{\text{)}}}^2}}} = 23.89 \cr} $$

Then, considering a 20% probability of attrition, a total requirement of 30 individuals was calculated. Sixty nurses who worked in the ICUs were recruited using purposive sampling. Unaware of the study process, a researcher assistant used a coin toss to assign the nurses into two groups: the training group utilizing the mHealth application (*n* = 30) and the control group (*n* = 30). The study’s inclusion criteria for participation were: willingness to take part in the study, a minimum of six months of experience working in the ICUs, possession of a smartphone, and no recent use of drugs that affect mental health or experiences of emotional crises within the past six months. Meanwhile, the exclusion criteria consisted of unwillingness to continue cooperation, non-use of the application, incomplete questionnaire responses, and facing a crisis during the study. All participating nurses provided informed consent and remained in the study until its completion, with no one withdrawing.

### Intervention

At first, an expert in resilience training with a Ph.D. in psychology provided resilience skills training to the research team. Then, a mHealth application titled “Resilience” was developed based on the micro-learning educational method. The application was designed for the Android platform on a smartmobile phone. The educational content of the application was developed by integrating resilience techniques from the comprehensive standards guidelines published by the Ministry of Health, Treatment, and Medical Education of Iran, as well as other literature [7, 22, 23]. A team comprising two experts with nursing doctorates, one expert with a psychology doctorate, and two psychiatrists confirmed the content validity of the application.

**Fig. 1:**
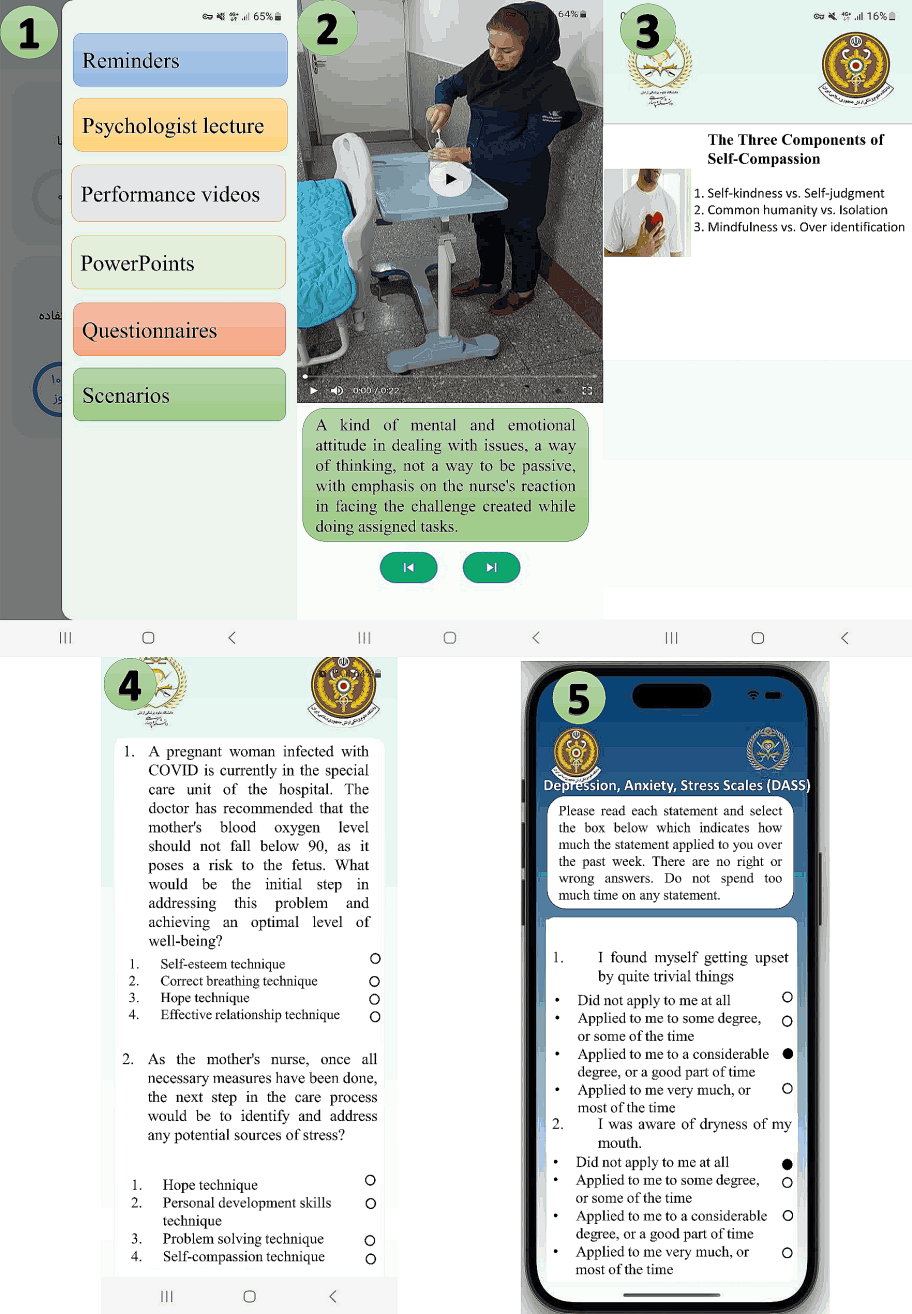
The resilience application is comprised of various components: (1) The main menu featuring main and sub-sections of the mHealth app that use micro-learning training (2), Practical demonstrations of resilience techniques (3), Voiced PowerPoints (4), The second scenario (5), The anxiety, and stress questionnaire

The training program for nurses commenced with an initial in-person gathering, during which the participants were formally introduced to the researchers. During this meeting, the research’s title, objectives, and methodology were explained, and the “resilience application” was introduced along with instructions on how to use it. The training on installation and operation of the application was provided to the nurses by a researcher who was a master’s degree student specializing in critical care nursing and possessed eight years of experience in several internal departments, as well as ICU and critical care units. The duration of this training, extending to five months, was influenced by several factors: dedicating time to familiarize participants with the software’s general menus, designing numerous questions within the virtual group to foster a deep understanding of the educational materials, and developing both random and detailed questions for the first and second scenarios, which required careful and repeated study of the materials. Moreover, a one-week gap was intentionally incorporated between the completion of application steps and the assimilation of the training materials, further extending the timeframe. Additionally, the ICU nurses are very busy and the researcher had to coordinate with them during their breaks or when they had nothing to do in the ward. The application also included pre-test and post-test assessments, which the nurses completed, and their results were sent to the researchers upon completion. The resilience program encompassed three main components: educational content, scenarios, questionnaires, and a subsection called reminders (Fig. 1). The training materials were presented in three formats: educational videos delivered by a psychologist, practical demonstration videos conducted by a clinical nurse, and voiced PowerPoints. The psychologist covered various topics during the training, including resilience, proper breathing techniques, self-esteem, self-compassion, hope, effective communication, problem-solving skills, and the development of advanced resilience skills. Educational design principles based on the micro-learning method were employed to develop the content, resulting in the creation of 3–5 min videos and multimedia materials (Fig. 1). In these videos, one of the researchers and a critical care nurse with five years of experience practically demonstrated resilience techniques to the nurses. Voiced PowerPoints were used as supplementary material for resilience training.

To prevent contamination bias, a user-password application was designed. Nurses in the intervention group were instructed not to share the educational materials with other nurses upon completion of the study. Once logged into the application, users could access the educational materials in a sequential order. To proceed to the next stage, users had to read all the files within each stage completely. Users could not adjust the speed of PowerPoint videos or audio files. Users received a reminder on their smartphones one week after completing all the educational content, followed by activating the first scenario. In the first scenario, users answered two multiple-choice questions based on the content they had learned. The second scenario (Fig. 1) was activated if both questions were answered correctly. However, if one or both questions were answered incorrectly, users had to revisit the previous stage and review the educational content thoroughly. This process continued until the activation of the second scenario. After completing the first scenario, users answered two new questions based on the previous training. If both questions were answered correctly in the second scenario, the stress and anxiety questionnaire would be activated for the user (Fig. 1). Otherwise, the user would be directed back to the previous step to review the educational content before proceeding. A virtual group was created on a social networking platform to address the nurses’ questions, and one of the researchers interacted with them. Reminders were sent to users every seven days to ensure their progress in completing the training. After thoroughly viewing the educational materials and accurately answering the questions for the two designed scenarios, the nurses proceeded to complete the questionnaires within the application. Their recorded responses were then automatically transmitted to a researcher via an email system integrated into the application.

### Data collection

In the designed mHealth application, questionnaires regarding personal characteristics, and stress and anxiety were included. These questionnaires were used in two stages: pre-test and post-test. Nurses completed these questionnaires at both stages, providing data for analysis.

The personal information questionnaire encompassed age, work experience in nursing, work experience in the ICUs, sex, education status, employment status, shift work, participation in the resilience workshops in the last six months, and experience caring for patients with COVID.

The Depression, Anxiety, and Stress Scale-21 (DASS-21), developed by Lovibond in 1995, was utilized to measure stress, anxiety, and depression. This questionnaire consists of subscales for depression, stress, and anxiety, each comprising seven items. The final score for each subscale is obtained by summing the scores of the corresponding items. Each item is rated on a scale from zero (not applicable to me at all) to three (extremely applicable to me). Items 21, 17, 16, 13, 10, 5, and 3 pertain to depression; items 20, 19, 15, 9, 7, 4, and 2 relate to anxiety; and items 18, 14, 12, 11, 8, and 6 are associated with stress. This study used the stress and anxiety subscales of DASS-21. The scores for the stress and anxiety subscales range from zero to 21. The reliability of this questionnaire has been reported in various studies conducted with the Iranian population and nurses, with Cronbach’s alpha coefficients of 0.87 [24, 25]. The validity of this questionnaire has been confirmed in both Iran [24] and other countries [26]. In Iranian studies, the test-retest validity for the stress and anxiety scale was reported as 0.8 and 0.78, respectively [24]. In this study, the Cronbach’s alpha coefficients for the stress and anxiety subscales were 0.78 and 0.79, respectively.

**Fig. 2:**
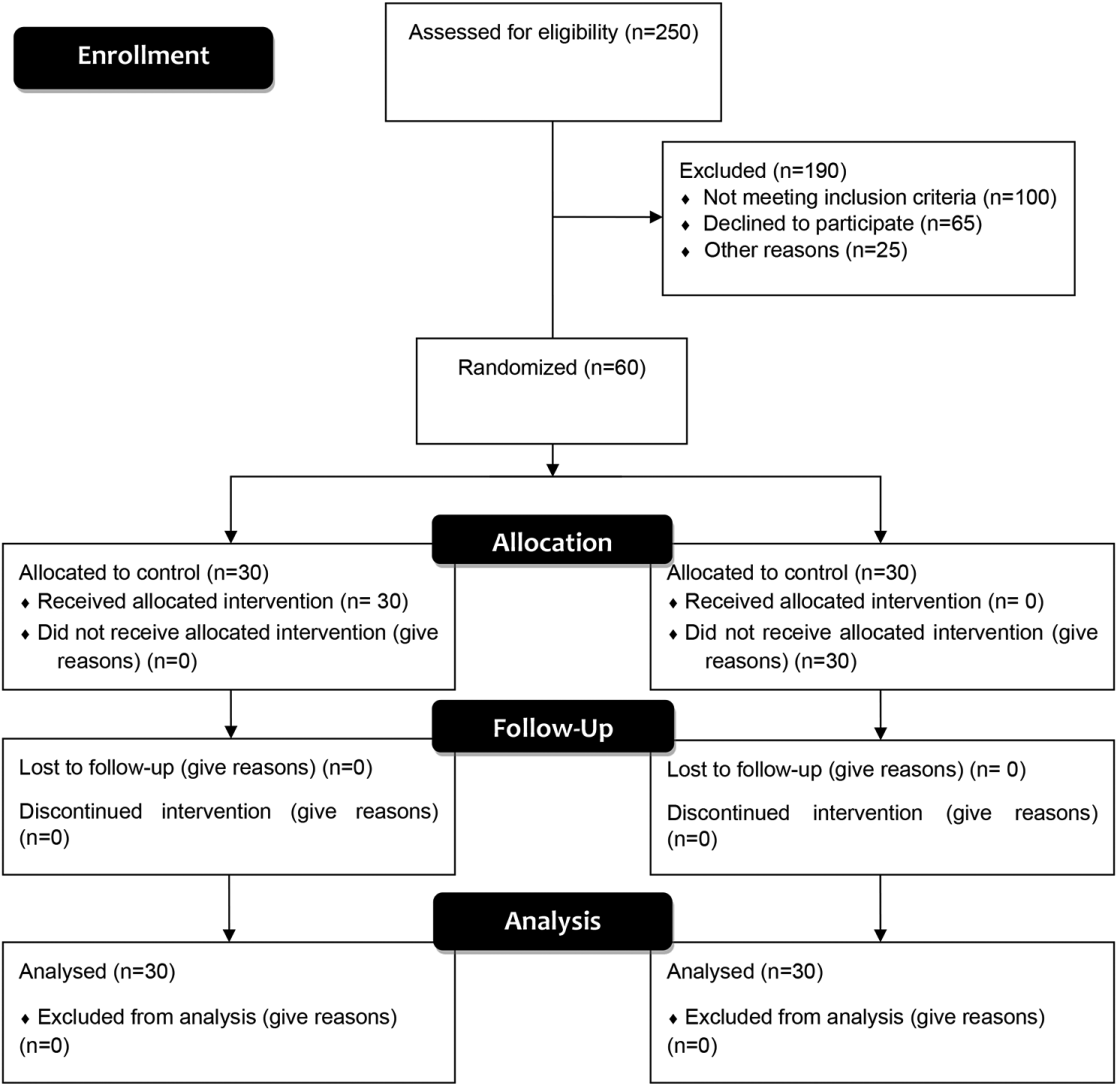
The study process

During the study, the researchers did not give training to the nurses in the control group. In addition to considering the user and password for the application, the data was first collected from the control group and then from the intervention group. The control group completed the pre-test and post-test in-person in the considered standard setting. The answers of the intervention group via application, incorporating the initial approximation determined by the researchers through embedded training and virtual group discussions, were gathered over five months. The responses from the both groups were received by the researchers in the fifth month. After completing the study, the application was also provided to the control group. The conduct of the study is shown in Fig. 2.

### Data analysis

The data analysis was performed using SPSS software, and descriptive statistical tests such as mean, SD, frequency, and percentage were employed. Several other statistical tests were utilized, including Fisher’s Exact Test, Chi-Squared Test, Independent *t*-Test, Paired *t*-Test, Mann–Whitney *u*, and Wilcoxon Signed Ranks Test. All assumptions for the statistical tests were met. The Kolmogorov-Smirnov test was applied to assess the normality of the data distribution. A significance level of *P* < 0.05 was considered for the variables. The statistical analyst was blinded to the allocation of nurses to the intervention and control groups.

## Results

The nurses participating in the study had a mean age of 29.58 ± 4.68 (22 to 43) years. Their mean working experience in the nursing profession was 9.21 ± 6.35 [1–29] years, and their experience in ICUs was 4.48 ± 3.87 [1–16] years. The participants in the study were mostly male (71.67%), and 56.67% held a bachelor’s degree. Most were permanently employed (53.34%) and predominantly worked night shifts (30%). A significant portion (73.33%) of the participants had not undergone resilience techniques training courses. Furthermore, 41.67% of the nurses reported less than six months of experience in caring for COVID. The intervention and control groups were statistically homogeneous in terms of individual characteristics (Table 1) (*P* > 0.05). The mean and SD of stress and anxiety scores among all participating nurses were 10.43 ± 2.81 and 9.27 ± 2.61, respectively.

**Table 1:** Individual characteristics of nurses in ICUs

Group			Intervention		Control		Test, value, df, *P*-value
Variable			Mean (SD)		Mean (SD)		
Age (y)			30 ± 5.47		29.16 ± 3.78		*t= -0.685 df = 58 *P* = 0.496
Work experience in the nursing (y)			10.53 ± 7.92		7.90 ± 3.96		*t= -1.628 df = 42.696 *P* = 0.111
Work experience in the ICUs (y)			5.20 ± 4.95		3.77 ± 2.20		*t= -1.447 df = 40.068 *P* = 0.156
Variable			f	%	f	%	Test value, df, *P*-value
Sex	Male	19		63.33	24	80	** *P* = 0.252
	Female	11		36.67	6	20	
Education status	Bachelor	17		56.66	17	56.66	**Value = 1.815 *P* = 0.483
	Master	13		43.34	11	36.66	
	Ph.D.	0		0	2	6.68	
Employment status	Contractual	9		30	4	13.33	***Value = 2.648 df = 2 *P* = 0.266
	Contract	6		20	9	30	
	Permanent	15		50	17	56.67	
Shift work	Morning	6		20	4	13.33	**Value = 4.104 *P* = 0.397
	Evening	2		6.67	6	20	
	Night	10		33.33	8	26.67	
	Morning and night	3		10	6	20	
	Rotating	9		30	6	20	
Participating in the resilience workshops in last six months	Yes	8		26.67	8	26.67	** *P* = 1.000
	No	22		73.33	22	73.33	
Experience caring for patients with COVID	Less than six months	15		50	10	33.33	** *P* = 0.295
	More than six months	15		50	20	66.67	

ICUs: intensive care units; SD: standard deviation; y: year; f: frequency

*Independent *t*-test; **Fisher’s Exact Test; ***Chi-Square Test

The results showed that the mean scores of stress (*P* = 0.731) and anxiety.

(*P* = 0.498) in the intervention and control groups did not differ significantly before the intervention, While there was a significant difference after the intervention (*P* < 0.05) (Table 2). The findings of this study showed that the stress and anxiety levels of the nurses in the intervention group decreased after the use of a micro-learning-based mHealth educational application (*P* < 0.0001) (Table 2). After the intervention, there was a significant difference in the mean score of total stress and anxiety observed in the control group.

**Table 2:** Comparison of the mean score of stress and anxiety between intervention and control groups in nurses of ICUs before and after the intervention

Group	Stage	Intervention	Control	Test, value, df, *P*-value	Cohens’d
Variable		Mean (SD)	Mean (SD)		
Stress	Pre-test	10.77 ± 3.33	10.10 ± 2.19	*U = 427.000 *P* = 0.731	0.90
	Post-test	9.00 ± 1.66	10.73 ± 2.15	**t = 3.496 df = 58 0.001 = *P*	
	Test, value, df, *P*-value	***Z=-3.766 0.0001 > *P*	***Z=-2.933 *P* = 0.002		
Anxiety	Pre-test	9.43 ± 3.35	9.10 ± 1.63	*U = 405.000 *P* = 0.498	1.69
	Post-test	7.93 ± 0.98	10.23 ± 1.65	*U = 95.500 0.001 > *P*	
	Test, value, df, *P*-value	***Z=-3.333 0.0001 > *P*	***Z=-3.794 0.0001 > *P*		

ICUs: intensive care units; SD: standard deviation; df: degree of freedom

*Mann–Whitney *u*; **Independent *t*-Test, ***Wilcoxon Signed Ranks Test

According to Cohen’s method, the total effect size of mHealth educational application on the nurses’ stress and anxiety were 0.90 and 1.69, respectively, indicating that these were large and desirable outcomes (Table 2).

## Discussion

This study aimed to investigate the effect of training resilience techniques on stress and anxiety levels among nurses working in ICUs, using a mobile mHealth application that utilizes the micro-learning method. The results of training with mHealth application based on micro-learning in the current study are compared with the new training methods that emerged during the outbreak of the COVID pandemic so that the quantitative and qualitative impact of the new method presented can be determined.

Before the intervention, no significant differences were observed in the mean stress and anxiety scores between the intervention and control groups. These groups were homogeneous in terms of stress and anxiety levels. The mean stress and anxiety scores among the nurses in this study were lower compared to other studies conducted during the COVID pandemic [2, 27, 28], including the study by Ganjeali et al. [2] on the stress and anxiety of nurses providing care for patients with COVID in two hospitals in Tehran. Although their study setting was similar to this study, the timing differed. Ganjeali et al. [2] conducted their study during the peak of the COVID pandemic when nurses were more likely to experience heightened stress and anxiety due to increased exposure to COVID patients.

In contrast, our study was conducted during the post-COVID period, when it was expected for nurses to experience lower levels of stress and anxiety. Our study occurred after the seventh wave of the COVID pandemic in Iran. In our study, nurses’ longer exposure to COVID patients can contribute to increased experience and resilience levels. Evidence indicates that previous exposure to epidemics can enhance nurses’ familiarity with major outbreaks and public health measures, resulting in increased adaptability and flexibility [29]. Building resilience among nurses following stressful events helps them to quickly adapt and alleviate the long-term effects of negative psychological experiences [29].

In this study, after the intervention and use of the mHealth resilience training application based on the micro-learning method, the stress and anxiety scores of the nurses in the intervention group decreased. Also, the findings showed that the stress and anxiety scores of the nurses in the intervention group were lower than those in the control group. In addition, the effect size of mHealth resilience training application on nurses’ stress and anxiety was very large. These results are consistent with the results of other studies that have conducted interventions to reduce the stress and anxiety of nurses [2, 13, 15, 30–38]. Various studies have mentioned many techniques to reduce stress and anxiety, including progressive muscle relaxation, biofeedback, diaphragmatic breathing, autogenic training, relaxation response, cognitive behavioral therapy, and educational programs.

Considering physical techniques to reduce stress and anxiety of ICU nurses, for example, it has been reported in a study that after the educational intervention of muscle relaxation, the stress of the intervention group decreased by 21.28% and anxiety by 29.01% compared to the beginning of the study [2]. In Ratanasiripong et al.’s study on 60 general nursing students, using the biofeedback tool for four weeks, stress and anxiety decreased by 15% and 27%, respectively [30]. Liu et al. showed that diaphragmatic breathing could reduce nurses’ anxiety in caring for COVID patients by about 10% [31]. Also, using the autogenic exercise method on 70 nursing students in Bengaluru, India, stress fell by 17% [32]. In Calisi et al.’s study, the relaxation response method led to a reduction in stress and anxiety in the intervention group [33].

Considering psychological techniques to reduce stress and anxiety of ICU nurses, in Duva et al.‘s study, following the resilience-boosting intervention with cognitive-behavioral therapy via the application, stress reduction rates of 2% and 8.4% were observed one week and three months post-intervention [34]. In the study by Trottier et al. on healthcare workers, after the online intervention of cognitive-behavioral therapy, a decrease in the average score of stress and anxiety was reported by 28% and 6%, respectively [35]. Kock et al. studied the effect of intervention with two different digital applications on separate samples by studying a randomized controlled trial on healthcare workers; in this way, the use of two types of applications reduced the average anxiety score by 24% and 3% compared to the state before the intervention [13]. The reason for the eight-fold impact of one of the applications is due to the complete content and easy-to-use method defined in the first application [13]. Mathijs Nijland et al. with the intervention of an educational application based on environmental images of intensive care unit nurses, achieved a 3.4% reduction in the average stress score [36]. With the implementation of a mindfulness training set consisting of an application, an online workshop, and a workbook for nurses by Schönfeld et al. the mean perceived stress score decreased by 1.2% compared to the pre-intervention state, but in the long term, its effect decreased [15]. The decrease in the effect of education in the long term shows that nurses work in stressful environments and need continuous educational interventions [15]. Magtibay et al. also reported that using a several-week stress management and resiliency training program in a web-based method reduced nurses’ stress and anxiety by 38% and 52% after 24 weeks [37].

The percentage of stress and anxiety reduction in some studies [37, 38] was higher than in the recent study. This difference can be attributed to the type of intervention and the time allocated for training nurses. In previous research, a few weeks of face-to-face training was assigned to nurses, which was longer than the present study. However, in the searched databases, no study was found on using micro-learning to reduce nurses’ stress and anxiety, which could compare the present study’s findings with other studies.

After the intervention, the mean score of total stress and anxiety was significantly different in the control group in this study. During the current investigation, it was discovered that within the hospital where the nurses in the control group were located, two changes took place following the initiation of the study and the completion of the first questionnaire by the nurses: (a) The previous head nurse of the ICUs retired, and as a result, the newly appointed head nurse had not yet established effective communication with the nurses in the ICUs at the time of the study. According to evidence, organizational support and individual characteristics such as self-efficacy, flexibility, problem-solving, and emotional endurance help nurses maintain resilience [39]. ICU nurses require both managerial and emotional support from supervisors, who can positively affect the resilience of nursing staff through appropriate leadership styles. Shivola et al. identified three fundamental roles of supervisors that can enhance the resilience of nursing staff: relational leadership, creating a supportive and safe work environment, and effective communication [40]. (b) The nurses’ salaries were negatively impacted due to not receiving the required subsidies (payment for nursing services) during the study, affecting the nurses’ level of job dissatisfaction. These factors could account for the changes observed in the stress and anxiety scores of the nurses in the control group during the post-test phase.

The current study illustrates that a mHealth educational application utilizing micro-learning proves to be effective in alleviating stress and anxiety among nurses in ICUs in the post-COVID era. Training courses following the micro-learning approach can be tailored to digital devices, including smartphones. Micro-learning encompasses five key learning methods: integrating into daily work routines, utilizing distance learning techniques, addressing the forgetting curve, extracting data to enhance educational and career programs continually, and fostering a balance of hard and soft skills among healthcare professionals. This method enhances the efficiency of the teaching and learning process for nurses [21], thereby offering the potential for enhancing their mental well-being.

### Strengths and limitations

To the best of the authors’ knowledge, this study is the first to utilize a self-designed mHealth application based on microlearning to enhance resilience and reduce stress and anxiety among nurses working in ICUs. This study faced a limitation in its implementation due to issues with the application on nurses’ mobile phones. The researchers initially overlooked the minimum version required for the Android operating system installed on nurses’ phones, causing problems during the intervention’s implementation. After facing operational issues during the intervention, the researchers had to adjust the application to be compatible with lower versions of the Android operating system. For several participants, because of the application’s resource-intensive nature, its performance varied slightly across different phone models, leading to differences in execution speed.

## Conclusions

This study showed that using resilience techniques training through mHealth, based on the micro-learning training method on mobile smartphone platforms, can effectively reduce stress and anxiety among nurses working in ICUs. Despite nurses’ numerous challenges in these units, adopting innovative educational approaches like practical applications can significantly contribute to their training and professional development. This method offers several advantages, including the visually appealing nature of mobile-based technologies, integrated reminders within the application, accessibility at any time and place, and the opportunity for continuous and repetitive learning until mastery is achieved. These factors are crucial in motivating nurses to pursue continuous learning, fostering longevity, skill preservation, and stability in knowledge acquisition. Additionally, integrating the micro-learning teaching method with chatbot applications based on artificial intelligence in the work setting further meets nurses’ daily needs. By utilizing chatbot applications, nurses can apply their acquired knowledge to real-world problems encountered in their work. It is recommended that additional interventions using this novel educational approach on larger and more diverse samples be conducted.

## Data Availability

The datasets used and analyzed during the present study are available from the corresponding author upon reasonable request.

## Abbreviations

ICU: Intensive care unit
COVID: Coronavirus disease
DASS-21: Depression, Anxiety and Stress Scale-21
